# A mapping review of research on gambling harm in three regulatory environments

**DOI:** 10.1186/s12954-018-0265-3

**Published:** 2019-02-08

**Authors:** David G. Baxter, Margo Hilbrecht, Cameron T. J. Wheaton

**Affiliations:** 1 Gambling Research Exchange Ontario, 55 Wyndham St. N., Suite 214A, Guelph, ON N1H 7T8 Canada; 2 Department of Recreation and Leisure Studies, University of Waterloo, Waterloo, ON Canada

**Keywords:** Bibliometric analysis, Mapping review, Gambling harm, Conceptual Framework of Harmful Gambling, Canada, Australia, New Zealand

## Abstract

**Background:**

Harmful gambling is a complex issue with diverse antecedents and resulting harms that have been studied from multiple disciplinary perspectives. Although previous bibliometric reviews of gambling studies have found a dominance of judgement and decision-making research, no bibliometric review has examined the concept of “harm” in the gambling literature, and little work has quantitatively assessed how gambling research priorities differ between countries.

**Methods:**

Guided by the *Conceptual Framework of Harmful Gambling* (CFHG), an internationally relevant framework of antecedents to harmful gambling, we conducted a bibliometric analysis focusing on research outputs from three countries with different gambling regulatory environments: Canada, Australia, and New Zealand. Using a Web of Science database search, 1424 articles published from 2008 to 2017 were retrieved that could be mapped to the eight CFHG factors. A subsample of articles (*n* = 171) containing the word “harm” in the title, abstract, or keywords was then drawn. Descriptive statistics were used to examine differences between countries and trends over time with regard to CFHG factor and harm focus.

**Results:**

Psychological and biological factors dominate gambling research in Canada whereas resources and treatment have received more attention in New Zealand. A greater percentage of Australia and New Zealand publications address the gambling environment and exposure to gambling than in Canada. The subset of articles focused on harm showed a stronger harms focus among New Zealand and Australian researchers compared to Canadian-authored publications.

**Conclusions:**

The findings provide preliminary bibliometric evidence that gambling research foci may be shaped by jurisdictional regulation of gambling. Countries with privately operated gambling focused on harm factors that are the operators’ responsibility, whereas jurisdictions with a public health model focused on treatment and harm reduction resources. In the absence of a legislated requirement for public health or harm minimisation focus, researchers in jurisdictions with government-operated gambling tend to focus research on factors that are the individual’s responsibility and less on the harms they experience. Given increased international attention to gambling-related harm, regulatory and research environments could promote and support more diverse research in this area.

## Background

### A multidisciplinary approach to gambling studies

Harmful gambling is a complex issue with a diversity of antecedents and resulting harms. Likewise, “gambling studies” is a multidisciplinary and specialised field. As such, it can be challenging to get a holistic view of the state of research in the field. This raises questions as to whether all facets of harmful gambling are being adequately investigated, and if gambling researchers, like other scholars, are sometimes unaware of the breadth of research because of a tendency to focus on journals associated with their parent discipline. It has been shown that most scientific disciplines are slower to cite articles from other disciplines than those within their own discipline, suggesting a delay in knowledge transfer across disciplines [1]. When researchers do cite articles from other disciplines, they are predominantly citing more closely related disciplines and generally overlooking developments in other parent disciplines [2].

Recognising the limitations of a single or dominant disciplinary focus when examining the complex issues that precede harmful gambling as well as the resulting harms, some effort has been made among the gambling studies community to increase the awareness of contributions from diverse perspectives. The *Conceptual Framework of Harmful Gambling, Revised Edition* (CFHG) [3], co-authored by a panel of international, interdisciplinary experts, outlines eight interrelated general and gambling-specific factors that form an internationally relevant framework of antecedents to harmful gambling. It is intended to reflect the current state of knowledge and guide future research through a multidisciplinary lens.

This study examines how the academic gambling literature published over a 10-year period aligns with the eight CFHG factors. By doing so, we demonstrate areas of gambling studies where research is limited, along with others that have received considerable attention from researchers in the past. This will create a point of reference for recent research in all factors of harmful gambling. Further, we extend the focus by identifying studies with harm as a central component to explore how this subset of publications aligns with CFHG factors, and then compare the distribution by CFHG factor to the broader gambling studies literature.

Recognising that research programmes are often influenced by or developed in response to the public policy environment, we focus on Canada, Australia, and New Zealand to allow for a greater variety of research perspectives that could reflect different issues, priorities, and needs. To our knowledge, this is the first bibliometric study to quantify research on all factors of harmful gambling and to examine the concept of “harm” across the literature.

### Conceptualising gambling harm

Considerable effort in gambling research has been directed towards people with problem gambling: the small percentage of the population who experience severe harm from gambling. Using an established measure such as the Problem Gambling Severity Index (PGSI), gambling behaviour is typically assessed along a risk continuum as non-problem, low-risk, moderate-risk, and problem gambling [4]. Higher levels of psychological distress, relationship damage, financial difficulty, and lower levels of well-being typically occur at the upper end of the continuum [5–7]. The most severe behaviour, gambling disorder, is currently the only behavioural addiction in the *Diagnostic and Statistical Manual of Mental Disorders* (DSM-5) [8]. At the general population level, the percentage of people with gambling problems is relatively low. In Australia, for example, those identified as problem gamblers constituted about 1.1% of the population in 2015, with much higher percentages in the moderate-risk (2.6%) and low-risk (4.2%) categories [9].

Gambling scholars are increasingly turning their attention to gambling-related harms experienced across the full spectrum of gambling behaviour and not just by people with problem gambling. Gambling-related harms affect individuals, families, and communities in multiple life domains, and can include long-term and intergenerational effects [10]. A team of Australian researchers [11, 12] has developed a methodology to estimate a relative weighting of the health-related quality of life decrements experienced by people with low-risk, moderate-risk, and problem gambling. Following this public health model, they found that 85% of gambling harm in Victoria, Australia was attributed to low- and moderate-risk gamblers [12]. This is because people with low- and moderate-risk gambling constitute a higher percentage of the population and, therefore, experience a greater percentage of the harm at the population level than the much smaller number of people with problem gambling. Therefore, it is important to not only consider characteristics and conditions that contribute to harmful gambling, but also how people at all levels of risk contribute to gambling-related harm.

### Harmful gambling and gambling-related harm

Within gambling studies, two complementary frameworks outline the antecedents to harmful gambling, and the types of harm that can result from gambling. Both draw upon well-recognised theoretical models and policy frameworks such as Korn and Shaffer’s seminal public health framework [13] to consider a variety of influences at individual, institutional, and societal levels.

The CFHG defines harmful gambling as “any type of repetitive gambling that an individual engages in that leads to (or aggravates) recurring negative consequences such as significant financial problems, addiction, or physical and mental health issues” ([3] p4). The degree of harm can vary from inconsequential to chronic, and harm can occur across the risk spectrum of gambling behaviour. The authors outline how the eight interrelated factors contribute to harmful gambling, and the state of knowledge for each factor. The framework includes four “general factors” (cultural, social, psychological, biological) and four “gambling-specific factors” (gambling environment, gambling exposure, gambling types, gambling resources).

Whilst the CFHG mostly examines antecedents to harmful gambling, Langham et al. [10] developed a taxonomy of harms focused on outcomes. The taxonomy is a complementary framework to the CFHG that classifies gambling harm into seven dimensions across three temporal categories (general, crisis, and legacy harms). Drawing on diverse methodological approaches, the dimensions related directly or indirectly to gambling include relationship disruption, financial, emotional, physical health, work performance, cultural, and criminal [10]. Many of the dimensions overlap with the CFHG factors, indicating a high degree of interrelationship between the two frameworks.

### Previous bibliometric studies of gambling research

There is a small number of previous bibliometric studies on gambling research outputs. Eber and Shaffer [14] examined 1277 gambling-related citations from bio-behavioural research published between 1964 and 1999. They categorised the citations into eight heuristically developed categories, with “Cognition or Personality” being the most prevalent. Shaffer et al. [15] expanded on this study with a keyword analysis of 2246 gambling citations from 1903 to 2003. Besides “gambling”, the most common keywords in the dataset were “pathological gambling”, “risk taking”, “decision making”, and “addiction”. In the subset of articles published from 1999 to 2003, there was increased focus on epidemiology, comorbidities, neuroscience, and demographics. The keyword analysis also suggested a divergence between “gambling studies” and “pathological gambling studies”, indicating new facets of understanding gambling. More recently, Moon et al. [16] performed a broad bibliometric and thematic analysis on 9128 gambling-related articles published from 1960 to 2016. This study found an increased emphasis on public policy and technology in the last 10 years. Another notable finding from this study is that different countries tend to produce research that is framed in one of the three regulatory models proposed by Kingma: The “Prohibition model” (gambling is a sin), the “Alibi model” (gambling is a vice), and the “Risk model” (gambling is entertainment) [17]. All three bibliometric studies found an emphasis on decision and risk in the gambling literature.

Although these studies provide a strong contribution to our understanding of the focus and development of gambling studies, Eber and Shaffer [14] and Shaffer et al. [15] are limited in scope because they used only the PsycINFO and MEDLINE databases, which are specific to the disciplines of psychology and medicine respectively. Moon et al. [16] frame their analysis around a regulatory model, which also constrains the breadth of literature that was included. A limitation of all three studies, as conveyed by Eber and Shaffer [14], is that the database searches do not exclude studies where gambling is not the object being investigated, but rather a means used to study another phenomenon (e.g. the Iowa Gambling Task used to study decision making unrelated to gambling). As such, research related to cognition, risk-taking, and personality may be overrepresented. Furthermore, none of these studies investigate research on gambling harm, a topic of growing concern in gambling research and regulation.

### Bibliometric studies of other multidisciplinary fields

In other multidisciplinary fields, bibliometric studies have been conducted to identify all journals where related work may be published (e.g. Drago and Kashian [18]), and methodologies have been proposed for defining the scope of a multidisciplinary research area for bibliometric analysis using subject headings [19, 20]. More recent reviews of multidisciplinary fields employ keyword searches of article databases using lists of keywords and sub-areas agreed upon by domain experts [21], or perform a very broad keyword search followed by a thematic analysis to determine the sub-areas [22].

### Applying harm frameworks to the multidisciplinary gambling studies literature

Although there have been decades of research on gambling harm, the field of gambling studies is still relatively new in terms of disciplinary development. There is no agreed-upon framework or subject heading system for conducting a straightforward thematic analysis. It is particularly difficult to define the scope of gambling studies not only because of the diverse antecedents to harmful gambling, but also because problem gambling is almost always associated with multiple, complex, and diverse comorbid disorders [23–26]. Earlier review methods proposed by Rogers and Anderson [19] rely on using *Medical Subject Headings* (MeSH), which, if applied to gambling, would not account for research outside the medical context. Whilst traditional disciplines have established vocabularies and subject headings to assess the scientific outputs of the field (e.g. MeSH for medicine, Chemical Abstracts Plus (CAplus) headings for chemistry), multidisciplinary fields have relied on thematic analysis to categorise the work (e.g. Gurzki and Woisetschläger [22]). The field of gambling studies does not have any recognised term list; however, the CFHG provides a solid framework with which to explore the scope of the field.

### Gambling environment, regulation, and research

This study aims to identify research on gambling harm that aligns with to CFHG factors across all disciplines in selected countries in order to better recognise the scope and foci of gambling studies. The CFHG recommends that publicly funded gambling research and evaluation should be independent from gambling revenue generation in order to create a “comprehensive, policy-oriented research agenda” ([3] p21). Recognising that research agendas are shaped in part by existing policies and practices and future policy goals, it is important to consider that researchers will focus on topics that are relevant to policies and practices of their own jurisdictions. In order to obtain a fuller picture of the work undertaken in gambling studies within the limited scope of this review, we examine the research output from three countries with different regulatory frameworks for gambling that have active gambling research programmes: Canada, Australia, and New Zealand. Each of these countries has adopted a sufficiently different policy approach to gambling that could play a role in shaping research priorities and interests.

### Canada

Gambling regulation in Canada is relatively restrictive for a country in which land-based gambling and lotteries are legal in all provinces and territories. Casino gambling and online gambling may only be operated by the provincial governments and Indigenous groups, and in some provinces, bars and lounges can receive a license from the provincial regulatory body to operate electronic gambling machines (EGMs) or video lottery terminals (VLTs) [27]. Federal legislation of gambling is limited to the Criminal Code of Canada [28], and therefore the regulation of gambling and policies to mitigate its harms are left entirely to the provinces. As of 2017, Canada has 4744 EGM/VLT venues including 77 casinos, and government-operated online gambling is available in 8 of 10 provinces [29]. The gross government-operated gambling revenue for 2016–2017, which also includes revenues from bingo and lottery ticket sales, was at least CAD 16.8 billion. Based on the most recent Census of Population data, this amounts to approximately CAD 597 per adult [29].

The most recent nationwide problem gambling prevalence rate for Canada was 2.0% in 2007, with substantial variation among provinces. Quebec had the lowest rate of 1.1% and British Columbia the highest at 2.8% [30]. Separate province-level prevalence surveys have been undertaken more recently with problem gambling rates ranging from 0.2% in Manitoba in 2017 to 1.0% in New Brunswick in 2014 [29].

Canada is a major contributor to gambling studies. In a worldwide literature scan, Canada ranked third in the number of gambling-related scholarly publications produced since 1960 [16]. From 2000 to 2013, the Ontario Problem Gambling Research Centre (OPGRC) was the world’s largest single funder of gambling research [31]. Currently, the Canadian Consortium for Gambling Research includes six active member organisations representing six provinces that collaborate to support major research projects in gambling studies [32].

### Australia

As in Canada, gambling in Australia is regulated by state and territorial governments, with the exception of online gambling, which is regulated nationally through the 2001 Interactive Gambling Act [33]; however, land-based gambling is operated very differently from Canada. With the exception of Western Australia, all states and territories allow EGMs to be operated in hotels and other entertainment venues. State governments issue gambling operation licenses to these venues, called “clubs”, where the gambling is operated privately [34]. As of 2014, Australia has 13 casinos, and approximately 4000 clubs held active gambling licenses [27]. Australia also has an active sports and race wagering industry. Wagering in Australian states is privately operated by monopoly organisations called Totalisator Agency Boards (TABs), which are licensed by state governments. In the 2014/2015 year, the total gambling expenditure was an estimated AUD 22.7 billion, or AUD 1240 per adult [35], and in 2015 the PGSI problem gambling prevalence rates were found to be 1.2% problem gambling and nearly 8% for any level of problem gambling risk [36].

The Australia Productivity Commission has performed major inquiries on the effects of problem gambling [34, 37], and from 2002 to 2014 and 2017 to present, a national gambling research programme called Gambling Research Australia operates with representatives from all state and territorial governments and the national government [38, 39]. The state and territorial governments also have their own research programmes; a prominent contributor is the Victorian Responsible Gambling Foundation, which has taken a public health approach to gambling harm in its current research agenda [40]. In response to the 2012 Gambling Measures Act, the Australian Institute of Family Studies started the Australian Gambling Research Centre (AGRC), whose research programme emphasises inquiry into the harm caused by gambling, and measures to reduce the harm [41]. Although gambling harm is the stated research priority of the AGRC, it is worth noting that the overall objective of the Gambling Measures Act is “to encourage responsible gambling by all gamblers” ([42], section 4).

### New Zealand

New Zealand has a unique model of gambling regulation. In 2003, the government of New Zealand passed the Gambling Act, which created a national regulatory body for gambling whose stated purposes are consistent with a public health approach [43]. It remains the only country whose national government recognises and approaches gambling as a public health issue, with harm minimisation, harm prevention, and public health promotion required in all problem gambling strategies. In the 2015/2016 year, the total gambling expenditure in New Zealand was NZD 2.209 billion or NZD 585 per adult. At the same time, the 2016 New Zealand Health and Lifestyles Survey indicated that the PGSI problem gambling prevalence rates among adults were 3.3% for low-risk, 1.5% for moderate-risk, and 0.1% for problem gambling [44].

The New Zealand Gambling Commission promotes gambling-related research, including an active gambling research programme at the Addictions Research Centre at Auckland University of Technology, and a current annual gambling research budget of NZD 1.99 million managed by the Ministry of Health [45].

To summarise, gambling studies is a multidisciplinary field with an emerging body of theoretical approaches to understanding harmful gambling and gambling-related harm. Although a small number of bibliometric studies of the gambling literature have been conducted, they mostly focus on problem gambling from a psychological perspective with much less attention given to gambling harm across a broader range of disciplines and across the behavioural risk spectrum. One of the challenges in conducting a bibliometric study of gambling studies publications has been the lack of a recognised subject list. Given the relative newness of this field when compared to more traditional disciplines, alternate approaches could be applied. The *Conceptual Framework of Harmful Gambling* is a comprehensive framework that uses a public health perspective to understand harmful gambling and complements the Langham et al. taxonomy of harms [10]. By aligning the literature with the eight CFHG factors, a more comprehensive picture could emerge of geographic and temporal trends in gambling studies. Further, the CFHG also allows comparison of areas of thematic interest. With the attention of gambling studies scholars and policy makers turning to gambling harm, an area of considerable interest is the distribution of the literature in relation to harm and how this is approached by academics working in different jurisdictions.

With this in mind, we review gambling studies publications over a 10-year period to identify framework factors where research is limited, explore trends over time in selected countries, and examine research foci related to gambling harms. More specifically, we address the following questions:

1. To what extent does gambling-related research published from 2008 to 2017 align with CFHG factors at national and sub-national geographic levels?
2. How have research emphases on the different CFHG factors changed over time?
3. How does this differ among countries with different regulatory models, i.e. Canada, Australia, and New Zealand?
4. To what extent does the research produced by the three countries explicitly examine harm, and how do harm-focused publications align with CFHG factors?

## Methods

This study is a mapping review of gambling studies academic literature published during the 10-year period of 2008 to 2017. A mapping review is similar to a scoping review, but instead of aiming to determine the scope of knowledge on a topic, the goal of a mapping review is to describe and categorise knowledge within a topic of known scope in order to identify gaps in the literature [46]. In this review, we use bibliometric methods to measure trends in the literature. “Bibliometrics” refers to any mathematic or statistical methods applied to books or other media [47]. Bibliometrics are well suited to evaluating scientific output and identifying publication patterns according to discipline, authors, or other factors [48]. Although bibliometric methods are often used to better understand impact through citation analysis, for this study, we are less concerned with impact measures and more interested in the extent to which the pattern of publications aligns with CFHG factors.

### Literature search

We selected the ISI Web of Science Core Collection database (WoS) to search for articles. This database is widely used for bibliometric analyses and has coverage in the sciences, social sciences, and humanities. WoS also allows for searching by author country, which was necessary for this study. A search was performed for articles that were published between the years 2008 and 2017, contained “gambl*” in the title, abstract, or keywords, and had an author from Canada, Australia, or New Zealand. Phrases known to be unrelated were excluded in the original search (i.e. “gamble’s solution”, “standard gamble”, “Proctor & Gamble”).

During the search process, we identified two gambling-specific journals in the WoS Emerging Sources Citation Index: *Journal of Gambling Issues* and *Gaming Law Review*. For these journals, we manually retrieved all relevant article references for years that were not indexed in the database.

### Inclusion/exclusion criteria

All research articles were included. Relevant meeting abstracts were included unless a corresponding research article was also found, so the study was not double-counted. Since “harm” is an area of current discussion, editorials and letters were also included. Book reviews, research protocols, conference proceedings, and issue introductions were excluded. Articles manually retrieved outside of WoS were assigned a document type based on WoS definitions.

All abstracts were reviewed to ensure relevance to the CFHG. Guided by Eber and Shaffer’s [14] concept of gambling as either the means or object of investigation, we only included articles where gambling was the object of investigation. Thus, we excluded articles that had gambling as the means of investigation only (e.g. employed the Iowa Gambling Task); only mention harmful gambling as a harm experienced by a study population; were related to gambling but fell outside the study of gambling harm or problem gambling (e.g. mathematics, hospitality and tourism, consumer analysis), or; were completely unrelated to gambling (e.g. author with surname “Gamble”).

### Categorisation to the *conceptual framework*

Two of the authors served as coders for categorising articles to the CFHG. All authors read the CFHG in full before beginning coding, and referred to the document regularly during the coding process. The eight inter-related CFHG factors are described below. For a thorough description of each factor, see Abbott et al. [3].

#### Gambling specific factors

1. Gambling environment (e.g. socioeconomic, public policy, and regulatory environment)
2. Gambling exposure (e.g. access to sites and venues, marketing and messaging, adaptation to new formats)
3. Gambling types (e.g. range of gambling activities, structural characteristics, gambling alone or with others)
4. Gambling resources (e.g. treatment interventions, service utilisation, prevention, and protection programmes)

#### General factors

5. Cultural (e.g. ethnicity, traditions, belief systems, gender)
6. Social (e.g. family and peer gambling involvement, neighbourhood, education, social demographics)
7. Psychological (e.g. personality and temperament, lifespan development, judgement and decision making, comorbid disorders)
8. Biological (e.g. neurobiology, genetic factors)

For each article, the coder read the title and abstract and assigned the article to one of the eight CFHG factors. Many articles examined complex issues that span multiple CFHG factors. In these cases, we paid special attention to the purpose of the study to determine the “primary factor” of the study. Additional factors and subfactors are not presented here but are available in the published dataset.

Some articles were directly related to the study of gambling and its harm, but could not be assigned to any of the eight CFHG factors. These included general gambling prevalence studies, discussions of new research methods, classification of problem gambling types, diagnosis of problem gambling, etc. These could be described as developments in ways of understanding, recognising, and measuring harmful gambling or gambling harm, but cannot be ascribed to the CFHG categories which describe antecedents of harmful gambling.

### Intercoder reliability

Before beginning coding in earnest, both coders independently classified random subsets of 15 to 30 articles and discussed the agreements and disagreements. This was repeated for four rounds until a desired level of agreement was reached (complete agreement on 14 of 15 articles, or 93.3%). When coders were unsure about an article, the article was marked and later reviewed by both coders. If there was disagreement, the third author weighed in for the final decision.

### Analysis plan

Since the research questions are exploratory in nature, descriptive statistics, consisting primarily of frequencies, cross-tabulations, and chi-square were considered appropriate and were calculated using SPSS v.25. For the geographic analysis, articles were assigned to the jurisdiction of the first author from Canada, Australia, or New Zealand by their institutional affiliation. This information comes from the “author address” information provided by WoS. If the first author was affiliated with multiple institutions in multiple jurisdictions within the three countries (e.g. S.N. Rodda affiliated with Monash University in Australia and Auckland University of Technology in New Zealand), the author was attributed only to the first institution listed. We employed categorical analysis to examine the differences in factors studied both across and within countries. Using geographic boundary files provided by the respective federal governments [49–51] and the QGIS software package [52], we created summary maps of research in the CFHG factors.

## Results

A total of 2293 citations were found in our original searches. After excluding articles unrelated to gambling studies or not from the target countries, 1574 articles remained. Of these citations, *N =* 1424 could be ascribed to a framework factor. Most publications were research articles (83.6%). The remaining publications represented reviews (6.6%), meeting abstracts (5.3%), editorials (3.2%), and letters (1.3%).

### Summary by country and subnational region

When country of the first author was examined, *n* = 750 (52.7%) originated from Canada, *n* = 612 (43.0%) from Australia, and *n* = 62 (4.4%) from New Zealand. Several articles (*n* = 60) included multiple authors representing two or more of these countries, but for these analyses we focus on the country of the first author only.

There were notable differences in the geographic distribution of publications within countries. This was anticipated based on the dispersion of research groups across each country. In Canada, more than 90% of the citations were from the provinces of British Columbia, Alberta, Ontario, and Quebec. In Australia, institutions in New South Wales, Victoria, and Queensland contributed more than 80% of citations, and in New Zealand, 8 of 10 publications were by researchers based in Auckland (see Table 1).

**Table 1:** Distribution of publications by national and sub-national geographic region (*N* = 1424)

Geographic location	*N*	%	Cumulative %
Canada			
Ontario	329	43.9	43.9
Quebec	164	21.9	65.7
Alberta	114	15.2	80.9
British Columbia	78	10.4	91.3
Nova Scotia	38	5.1	96.4
Manitoba	15	2.0	98.4
Saskatchewan	6	.8	99.2
New Brunswick	4	.5	99.7
Newfoundland and Labrador	2	.3	100.0
Total	750	100.0	
Australia			
New South Wales	215	35.1	35.1
Victoria	186	30.4	65.5
Queensland	94	15.4	80.9
South Australia	68	11.1	92.0
Australian Capital Territory	22	3.6	95.6
Northern Territory	16	2.6	98.2
Western Australia	10	1.6	99.8
Tasmania	1	0.2	100.0
Total	612	100.0	
New Zealand			
Auckland	51	82.3	82.3
Wellington	5	8.1	90.3
Canterbury	3	4.8	95.2
Otago	2	3.2	98.4
Waikato	1	1.6	100.0
Total	62	100.0	

### Summary by *Conceptual Framework* factor

Overall, psychological factors dominated the focus in the gambling studies literature at 32.7% of publications (see Table 2). Gambling resources received the second most attention (14.5%), with gambling exposure and social factors each represented in about 1 in 10 publications (9.3% and 11.5%, respectively). Factors that aligned with fewer than 10% of the publications included biological factors (9.8%), gambling environment (8.3%), gambling types (7.5%), and cultural factors (6.3%).

**Table 2:** Percentage of publications by country and framework factor

Country	Framework factor (%)							
	Gambling environment	Gambling exposure	Gambling types	Gambling resources	Cultural factors	Social factors	Psychological factors	Biological factors
Canada	4.8	5.2	6.9	12.8	4.4	12.3	39.9	13.7
Australia	11.8	14.4	8.0	15.7	8.0	10.9	25.5	5.7
New Zealand	16.1	8.1	9.7	24.2	12.9	8.1	17.7	3.2
Total	8.3	9.3	7.5	14.5	6.3	11.5	32.7	9.8

Each country showed a different pattern of alignment with framework factors. For Canada, the psychological factor was most often ascribed (39.9%). Biological, gambling resources, and social factors were represented at similar rates (13.7%, 12.8%, and 12.3%, respectively). Factors that received considerably less attention during the 10-year time frame when compared to the overall average were gambling types (6.9%), gambling exposure (5.2%), gambling environment (4.8%), and cultural (4.4%) (see Table 2).

For Australia, publications were more evenly distributed across the eight factors, and the distribution aligns more closely with the distribution of the overall sample. The psychological factor accounts for one quarter of all publications (25.5%). Social factors at 10.9% and biological factors (5.7%) are less than the total percentage for all countries, whereas gambling resources (15.7%) and gambling environment (11.8%) are somewhat more. The most similar were gambling types and cultural factors (both at 8.0%). The most notable difference is for gambling exposure (14.4% versus 9.3% of overall total) (see Table 2).

Although the number of publications from New Zealand was much lower than either Canada or Australia, a pattern emerged that was considerably different from the other two countries. Gambling resources was the most commonly ascribed factor (24.2%), which is perhaps not surprising given the public health focus on gambling in New Zealand. Psychological factors accounted for considerably fewer publications (17.7%) than in other countries, and had a level of interest similar to gambling environment (16.1%). The cultural factor was ascribed to 12.9% of publications, which was higher than the combined percentages of the other two countries. Gambling types (9.7%) also received more attention than in other jurisdictions. Gambling exposure and social (8.1% each) and biological factors (3.2%) had lower than average percentage attributed to these factors (see Table 2).

**Fig. 1:**
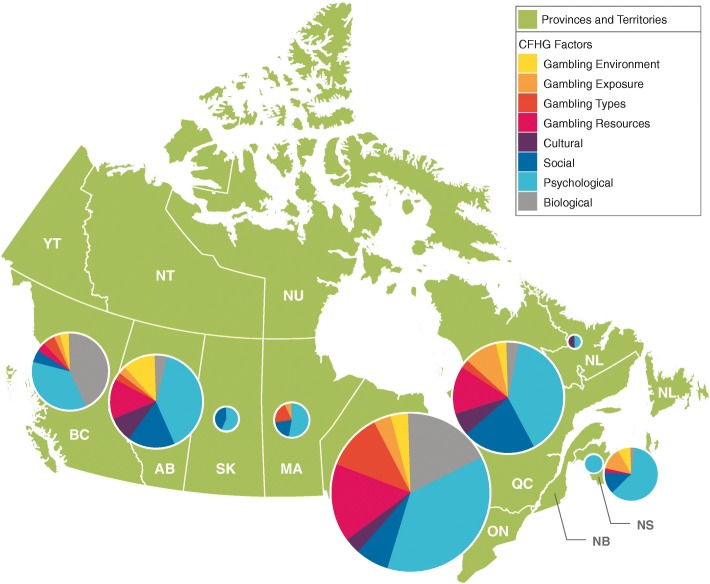
Number of publications by province and framework factor (Canada). Distribution of Canadian gambling research articles from 2008 to 2017 (*n* = 750), divided by province of first Canadian coauthor. The area of each pie graph is proportional to the number of articles from the province

The factors were then mapped to subnational geographic regions to determine where there was a stronger focus on specific framework areas. Figure 1 shows the distribution of publications in Canada by province and framework factor. The main centres for gambling research are located in Ontario, Quebec, Alberta, British Columbia, and Nova Scotia. In each of these provinces, about 4 in 10 publications align with the psychological factor. The exception is Nova Scotia, where more than 6 in 10 publications (60.5%) align with this factor. Biological factors were highly represented in British Columbia (43.6%). Most gambling resources publications originated in Alberta, Ontario, and Quebec. The provinces of Nova Scotia, Saskatchewan, Alberta, and Quebec had a greater percentage of publications ascribed to social factors when compared to Ontario.

**Fig. 2:**
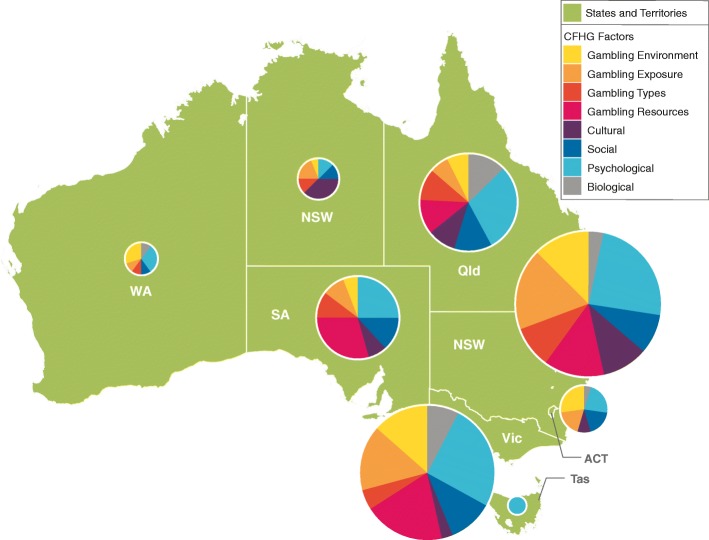
Number of publications by state/territory and framework factor (Australia). Distribution of Australian gambling research articles from 2008 to 2017 (*n* = 612), divided by state/territory of first Australian coauthor. The area of each pie graph is proportional to the number of articles from the state/territory

Australian publications were concentrated in the states of New South Wales, Victoria, Queensland, and South Australia (see Fig. 2). Within these states, at least one quarter of publications could be attributed to the psychological factor. South Australia had the highest percentage of publications assigned to gambling resources (29.4%). The Australian Capital Territory had a strong focus on the gambling environment (27.3%), perhaps not surprisingly as the seat of national government. On the other hand, the Northern Territory produced a higher proportion of publications related to cultural factors (37.5%).

**Fig. 3:**
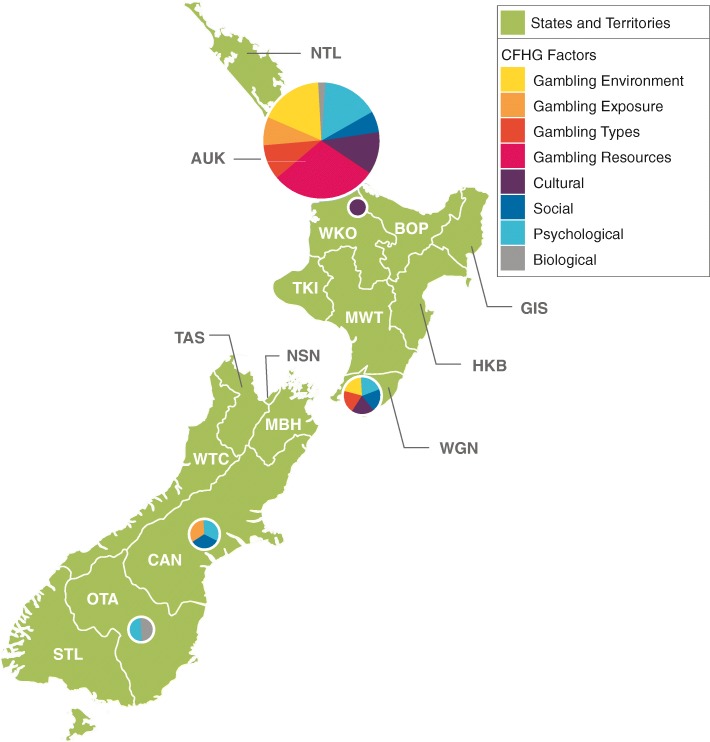
Number of publications by region and framework factor (New Zealand). Distribution of New Zealand gambling research articles from 2008 to 2017 (*n* = 62), divided by region of first New Zealand coauthor. The area of each pie graph is proportional to the number of articles from the region

Almost all New Zealand publications were based in Auckland, and about one third were attributed to the gambling resources factor (see Fig. 3). Because Auckland dominates the research landscape in New Zealand, the percentages of publications for each factor are very close to those for the country as a whole. One exception is the social factor, where Wellington and Canterbury each have a higher proportion of publications in this area. As can be seen in Fig. 3, smaller regions such as Canterbury, Otago, and Waikato have much more limited numbers of publications which, in turn, affect the coverage of framework factors overall.

### Change over time

**Fig. 4:**
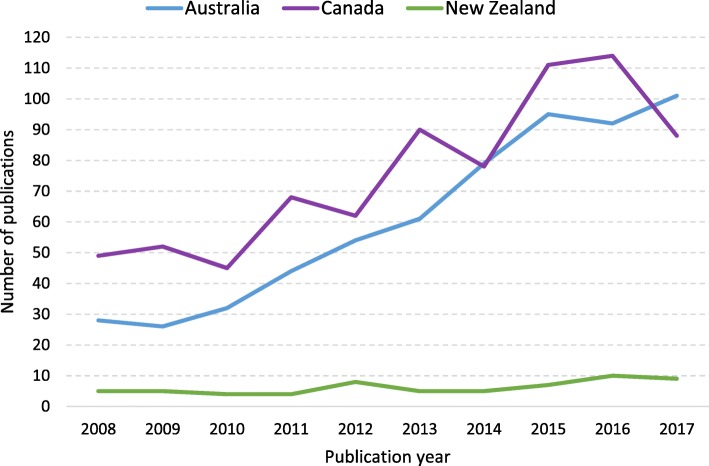
Total gambling studies publications mapped to the CFHG by country and year, 2008 to 2017

During the 10-year period of 2008 to 2017, there was steady growth in gambling publications generally. For both Australia and New Zealand, the increase over time was a relatively steady. Compared to 2008, the number of publications in 2017 was more than triple for Australia and double for New Zealand (see Fig. 4). Canada showed a more erratic pattern with periods of increase followed by periods of decline. In 2017, there was a substantial decrease in the number of Canadian authored publications. Overall, the relative number of publications produced each year by each country was similar and varied little over time [*χ2* (18, *N =* 1424) = 21.98, *p* = .233).

**Fig. 5:**
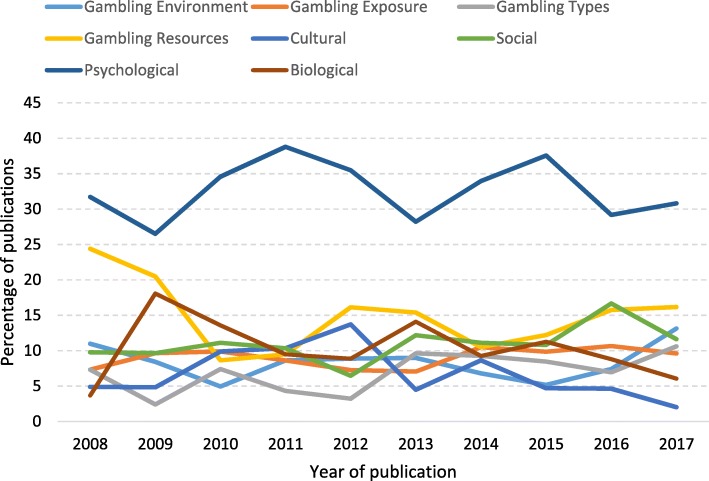
Number of gambling studies publications mapped to each CFHG factor by year, 2008 to 2017

When publications were examined by CFHG factor over time, the most obvious pattern overall was the dominance of the psychological factor (see Fig. 5). It accounted for about one third of publications each year, ranging from a low of 26.5% in 2009 to a high of 38.8% in 2011. In 2017, as in 2008, it accounted for just over 30% of publications. Other patterns were less obvious, and all factors showed some variation over time. For example, gambling resources, the next most common factor, was ascribed to about one quarter of the publications in 2008, dropped substantially in 2010 to 8.6%, and gradually increased to 16.3% in 2017. Cultural factor publications also showed an overall decrease. For all other factors, there was a small increase in the percentage of publications from 2008 to 2017.

When Canada was examined separately, there was little change in research emphases by framework factor over time [*χ2* (63, *N =* 750) = 68.18, *p* = .306). The psychological factor was ascribed to about 4 in 10 publications (39.9%), ranging from a low of 25.0% in 2009 to a high of 48.4% just 3 years later in 2012. There was a substantial decrease from 2008 to 2017 in publications related to gambling resources (from 26.5% to 14.9%), gambling environment (10.2% to 3.4%), and gambling exposure (from 6.1% to 4.6%). Biological factors increased from 6.1% in 2008 to 9.2% in 2017, gambling types increased by half from 6.1% to 9.2%, and cultural factors more than doubled from 2.0% to 4.6%. Social factors remained stable.

In Australia, there was a significant change in framework factor focus during the 10-year period [*χ2* (63, *N =* 612) = 98.86, *p* = .003). The psychological factor still dominated overall, but dropped from a high of 34.4% of publications in 2010 to 22.0% in 2017. Gambling resources, the next most commonly observed factor, also decreased from 21.4% in 2008 to 17.0% in 2017. On the other hand, gambling exposure increased from 7.1% in 2008 to 13.0% in 2017, gambling types from 7.1% to 11.0%, and gambling environment from 14.3% to 21.0% during the same time period. Like Canada, there was considerable variation among some factors over time. For example, the percentage of cultural factor publications began at a relatively low 7.1% in 2008, peaked at 20.4% in 2012, and then dropped to zero publications in 2017. Social and biological factors have remained relatively stable, increasing by two and three percentage points, respectively, over time.

New Zealand was excluded from year and framework factor analyses because low cell counts over the 10-year period precluded meaningful results.

### Harm-focused articles

Of the *N* = 1424 articles included in the dataset, 171 (12.0%) mentioned “harm” in the title, keywords, or abstract. New Zealand had the highest percentage of harm-focused publications (21.0%), followed closely by Australia (19.0%), and Canada (5.6%). The difference between countries was significant [*χ2* (2, *N =* 1424) = 61.80, *p* < .001) and likely reflects the policy and research shift towards public health and harm minimisation perspectives in New Zealand and Australia.

**Fig. 6:**
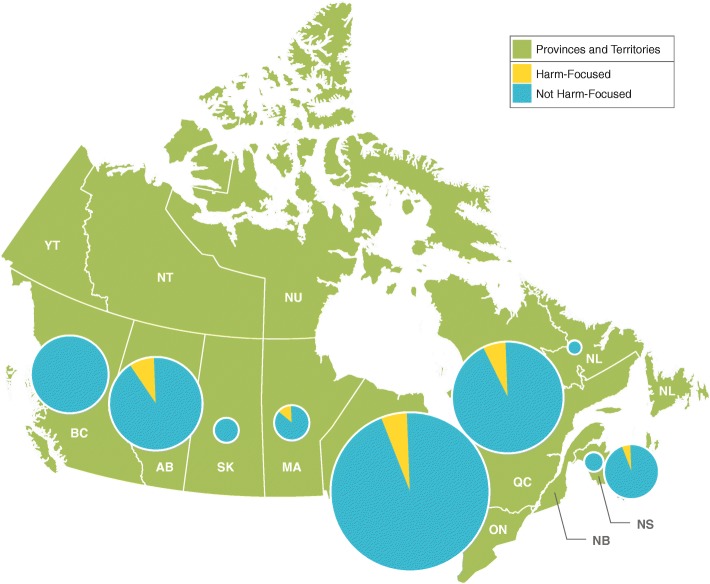
Percentage of Canadian publications with a harm focus by province, 2008 to 2017. Distribution of Canadian gambling research articles from 2008 to 2017 (*n* = 750), with percentage of harm-focused articles indicated. Articles containing the word “harm” in the title, abstract, or keywords were considered harm-focused. The area of each pie graph is proportional to the number of articles from the province

When examining the geographic dispersion of harm-focused publications at the subnational level, only five Canadian provinces had publications that met the inclusion criteria (see Fig. 6). Of these, the highest percentage was in Manitoba (13.3%), and the lowest in Nova Scotia (5.3%). Fewer than 1 in 10 publications based in the other provinces had a harm focus (Ontario, 5.2%, Quebec, 6.7%, and Alberta, 8.8%). It should be noted that the latter provinces produced substantially more publications overall than the former two (see Fig. 6).

**Fig. 7:**
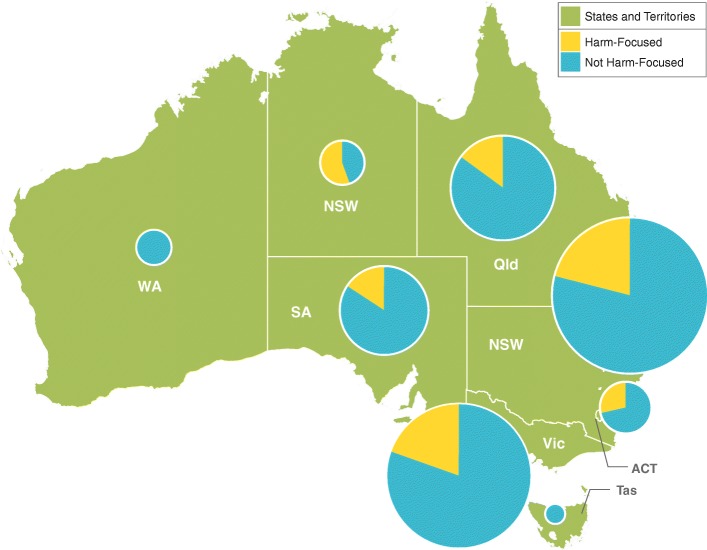
Percentage of Australian publications with a harm focus by state/territory, 2008 to 2017. Distribution of Australian gambling research articles from 2008 to 2017 (*n* = 612), with percentage of harm-focused articles indicated. Articles containing the word “harm” in the title, abstract, or keywords were considered harm-focused. The area of each pie graph is proportional to the number of articles from the state/territory

In Australia, six of eight states and territories had harm-focused publications. Notably, more than half of publications produced in the Northern Territory (56.3%) had a harm focus (see Fig. 7). In the larger research centres, about one in five publications was harm-focused (e.g. 19.5% in New South Wales, 21.0% in Victoria, and 22.7% in the Australian Capital Territory). The other states and territories were less well represented but still contributed more than most Canadian provinces (e.g. 12.8% in Queensland, and 13.2% in South Australia).

**Fig. 8:**
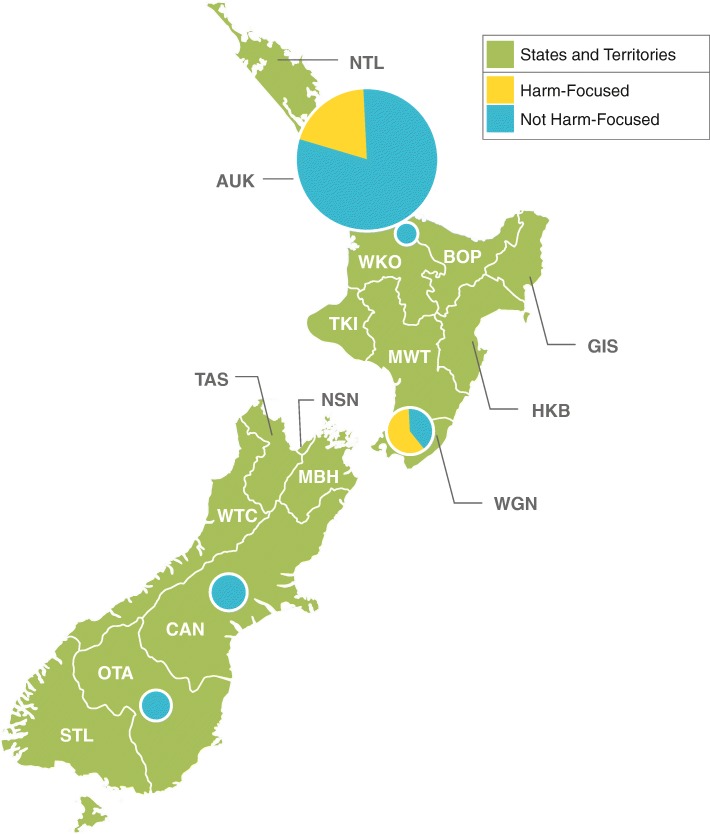
Percentage of New Zealand publications with a harm focus by region, 2008 to 2017. Distribution of New Zealand gambling research articles from 2008 to 2017 (*n* = 62), with percentage of harm-focused articles indicated. Articles containing the word “harm” in the title, abstract, or keywords were considered harm-focused. The area of each pie graph is proportional to the number of articles from the region

The centres in New Zealand produced a substantial proportion of harm-focused publications. In the largest region, Auckland, one in five publications (19.6%) had a harm focus (see Fig. 8). In Wellington, a much smaller centre, three in five publications had a harm focus (60.0%).

Overall, the number of harm-focused publications has been steadily increasing. Beginning with a low of 6.1% in 2008, it reached 15.3% in 2012. After a dip from 2013 through 2015, the number of harm publications began increasing again so that it represented 23.5% of publications in 2017. This upward trend may be expected to continue because of the increasing emphasis on understanding gambling-related harm and the continuing interest in harmful gambling among gambling studies researchers.

Despite the recent advances, only Australia had enough harm-focused publications to examine trends over time. Although there was variation during the 10-year time period, the trend was mostly upward. In 2008, only 7.1% of articles met the inclusion criteria. Five years later in 2012, the number had tripled to 22.2%. Although there were some declines along the way, in 2017, 32.0% of Australian publications had a harm focus.

Harm-focused publications were also analysed overall by framework factor. More than one quarter were ascribed to gambling exposure (27.3%), which is consistent with the factor’s focus on gambling setting, accessibility, and marketing and messaging. Gambling environment, with an emphasis on public policy, economics, the socio-political environment, and social responsibility accounted for 19.5% of harm-focused publications. This was followed by cultural factors (16.7%), gambling resources (15.0%), gambling types (14.0%), and social factors (12.2%). Psychological factors and biological factors were assigned least often to harm-focused publications (5.8% and 2.9%, respectively).

Again, due to low numbers of publications in the other two countries, only Australia allowed meaningful analyses by framework factor. Consistent with the overall pattern, most of the harm-focused publications were ascribed to gambling exposure (33.0%), followed by an almost equal percentage of gambling environment (25.0%) and cultural factors (24.5%) publications. Gambling types also had higher than average numbers of harm publications (22.4%), as did gambling resources (18.8%), psychological (10.3%), and biological factors (11.4%). Social factors (11.9%) were very close to the overall average.

## Discussion

### Research trends in *Conceptual Framework* factors

This was the first bibliometric study on gambling research to categorise publications using a framework developed within the gambling studies field. Although the CFHG was not originally created with the intent to categorise and map research ([3], p.6), it proved to be a useful framework for assessing areas where there has been considerable attention, and others where less research has occurred. As our bibliometric method proved successful using an existing typology, this shows promise for using this review method to investigate other trends in gambling research.

Consistent with the three previous bibliometric studies, our sample was dominated by articles that aligned with the psychological factor. Our sample was the first to find articles on gambling resources to be strongly represented, which demonstrates advancements in the field towards applying knowledge of gambling to reduce harm. Conversely, research on gambling types, cultural factors, social factors, and biological factors are least represented in the sample. To a degree, the difference in numbers represents disciplinary norms: some disciplines, such as anthropology or sociology, are more likely to use methodologies that may be more time consuming and generate fewer articles (e.g. an ethnographic study of cultural factors may take considerably longer than a secondary data analysis of a health survey associated with psychological factors).

Eber and Shaffer [14] note that judgement and decision-making research may be overrepresented in bibliometric investigation of gambling because the sample will include psychological studies that employ gambling tasks but are otherwise unrelated to gambling studies. In the present study, we manually removed all items unrelated to gambling studies, and we still found psychological research generally, and judgement and decision-making specifically, to dominate. Our findings provide bibliometric evidence for the criticisms of gambling research in the *Fair Game* report, where interviewees described gambling journals’ editorial boards as being dominated by individuals who encourage research focused on “problem gamblers” from psychological and medical perspectives [53].

The specific focus on judgement and decision-making highlights the preoccupation in gambling research with the individual’s decision to gamble. This perspective was articulated in 2004 as the Reno Model of responsible gambling, which “rests upon two fundamental principles: (1) the ultimate decision [to gamble] resides with the individual and represents a choice, and (2) to properly make this decision individuals must have the opportunity to be informed” ([54] p. 311). The Reno Model has been criticised for its narrow focus on individual responsibility that minimises the duty of care of governments and the gambling industry, and for industry “player education” initiatives excluding crucial information about the addictive nature of gambling products [55].

The focus on individual responsibility in gambling harm reduction programmes has been criticised for implying personal power when it is a lack of power that characterises the loss of control over one’s gambling [56]. Reith has also argued that the neoliberal emphasis on freedom of choice, informed choice, and personal responsibility in gambling reflects the wider socioeconomic context of Western societies [56]. Recent empirical research has found that government and industry discourses in Australia emphasise that individuals must exercise their self-control and monitor themselves in order to prevent gambling harm [57]. Further, these discourses may have a stigmatising effect on people experiencing harm, as they reason that if they were not “responsible”, then they must be irresponsible and lack self-control [58].

Although our sample was the first to find “gambling resources” to be strongly represented, it is possible that the resources research is primarily focused around individuals and their decisions to gamble. An increase in resources research will likely lead to gambling resources being implemented more effectively, and thus a reduction in gambling harm, but if these resources are predominantly framed around individuals with problem gambling, then other harms such as family harms and legacy harms remain unaddressed. We may expect a similar pattern at the public policy level: evidence-based policies to address gambling harms will primarily target individual harms and problem gambling if that is the type of evidence that is readily available. Policies framed around freedom of choice and personal responsibility may reflect larger neoliberal trends beyond the study and regulation of gambling.

### Research foci between countries

The CFHG was also effective for showing differences in research foci between countries and between states and provinces within countries. In the case of Australia, it also showed a change in research focus over the 10-year period, with an increased focus on gambling environment, exposure, and resources in later years. In New Zealand, articles on gambling resources, gambling environment, and cultural factors are most prominent, which aligns with the country’s public health approach to gambling harm. Articles from Australia were the most evenly distributed among CFHG factors and, of the three countries, had the highest proportion of gambling exposure research. Although advertisement of EGM gambling has been banned in Australia, EGM venues are still widely accessible and may be of concern. However, this advertisement ban does not apply to wagering on sports and racing, popular gambling products that are privately operated, heavily advertised, and widely accessible both online and offline. The high proportion of gambling exposure research in Australia may reflect broader concerns about the accessibility, marketing, and normalisation of these forms of gambling.

Canadian research was found to be the most concentrated, with much larger proportions of psychological and biological factor articles than the other countries. This may be in part due to institutional specialisations, such as the neuroscience research programme at University of British Columbia, but it also reflects how gambling research has been funded in the provinces historically. For example, from 2000 to 2013, the Ontario Problem Gambling Research Centre (OPGRC) committed over half of its total research funding to research on the assessment, treatment, and prevention of problem gambling, and the biological and psychological factors of problem gambling [31]. Although the OPGRC research reports were not included in the present analysis, its research priorities are still evident in the journal articles published by OPGRC-funded researchers.

It would be of interest to monitor the recent decline in numbers of Canadian publications to see if it continues. The decline was not seen for the other two countries, even though not all publications assigned to 2017 may have been available at the time of writing. It could possibly indicate the effect of more limited funding opportunities for gambling research in Canada. For instance, in 2013 the OPGRC, an Ontario government-funded organisation, experienced a change in mandate from funding gambling research to one of knowledge translation and exchange and was no longer able to support larger research projects [59]. At the same time, OPGRC’s name was changed to Gambling Research Exchange Ontario (GREO) to reflect the new directive. In short, there may simply not be as much research taking place as was the case in previous years. The concentrated nature of Canadian gambling research and overall decline following the mandate change of OPGRC suggest that the available research funding bodies and their research priorities have a considerable effect on the volume and nature of research that is pursued within a jurisdiction.

When examining harm-focused papers, there were clear differences between countries, with Canada having far fewer harm-focused papers, and New Zealand producing the most, proportionately. This may reflect the New Zealand Gambling Act’s requirement that the integrated problem gambling strategy, including nationally funded gambling research, focused on public health and harm minimisation ([60], section 317). However, for all three countries there was a positive trend over time in proportion of harm-focused papers, from 6% in 2008 to 21.5% in 2017. In their review, Shaffer et al. [14] observed a split between “gambling studies” research and “pathological gambling studies” research in the early 2000s. The increasing focus on harm in our sample suggests a continued maturation of gambling studies beyond this first split. The concept of “harm” is a subject of lively debate in gambling studies, with many recent developments in conceptual models of gambling harm [10, 12, 61].

In line with the limitations of decision-making research as a dominant means of addressing gambling harm, we expect that an explicit focus on harm in gambling research is likely to result in a more holistic approach to reducing gambling harm, since many people besides those with problem gambling experience gambling harm. In particular, New Zealand has been a leader in implementing and evaluating novel harm reduction programmes for gambling that include a community-action-based approach that reaches culturally diverse populations [62], and a programme that directs social directives such as gambling policies for workplaces and municipal governments [63]. Both of these programmes were created and evaluated in response to the New Zealand 2003 Gambling Act [62, 63].

However, as a result of the complexity of gambling harm, it is limiting to rely exclusively on the presence or absence of the word “harm” to investigate gambling harm research, as is it defined and measured differently by different researchers. Although we have not systematically analysed the meaning of “harm” in each of the harm-focused articles, gambling harm can be conflated with disordered gambling [64], and is often accounted for using limited measures such as problem gambling prevalence rates, PGSI scores [4], or the total consumption model [65].

### Limitations and future work

The CFHG proved to be a useful framework for mapping gambling research, although it does have some limitations. The framework was not developed for the purpose of categorising research, and as such, 9.7% of relevant articles could not be ascribed to a framework factor. Still, more than 90% could be assigned, which suggests that the framework is relatively comprehensive. Articles that could not be categorised fell under a few main themes, including research methods, screening methods, and problem gambling prevalence studies. Prevalence studies are of particular interest as they are a major piece of gambling research and yet their value has been recently called into question [66]. Future research could trace the prominence of prevalence studies over time.

Research articles investigate complex topics that often cover more than one factor of the framework. In the case that multiple factors were represented in an article, we recorded multiple factors but assigned one as the “primary” factor. Although secondary factors were not presented in this study, they were recorded for approximately one third of publications in our sample. Future analyses of this or other datasets could investigate trends in secondary factors. This would be especially revealing for articles assigned to the factor “gambling resources”, since this factor does not describe antecedents to harmful gambling, but is a general category for protective measures related to all other factors.

Although this exploratory mapping review allows greater insight into the gambling studies research landscape, it was limited to publications indexed in the WoS database. WoS is the largest single database of indexed scientific citations, but future work could include other academic databases to provide better coverage of the social sciences and humanities. Furthermore, since WoS only indexes research published by academic and commercial publishers, other sources of evidence in the “grey literature”, such as research reports published by governments or non-governmental organisations, were excluded regardless of their quality. In this context, grey literature refers to documents produced by governments, academics, business, and industry that are of sufficient quality to be collected and preserved, but not controlled by commercial publishers [67].

Grey literature is a valuable addition to review articles because its inclusion reduces publication bias [68]. Although grey literature searching is intensive, methods have been developed to systematically review grey literature to inform policy for public health programmes [69, 70]. In an applied, policy-oriented domain like gambling studies, government reports would more directly represent the policy agendas of their jurisdictions. Some of this work has already been done for Australia in the Productivity Commission’s 2010 report, which compiled ten years’ worth of government reports to examine the overall focus of government research and compare the foci between states [34]. Whilst some of the trends in Australian government-commissioned research are reflected in the current study, there are also some notable differences. For example, the Productivity Commission report found that New South Wales government research was heavily focused on counselling and support services (i.e. “gambling resources” in the CFHG), and that Queensland government research was focused more on the nature and extent of gambling and less so on the impacts of gambling and potential harm minimisation programmes (i.e. low “gambling resources” and low “harm-focused” in our typology) [34]. Neither of these trends is reflected in the current study. The value of grey literature has sometimes been mischaracterised and dismissed in review articles of gambling (e.g. Ladouceur et al., [71]), but this demonstrates how grey literature can provide a more complete understanding of the state of evidence. Future reviews of the gambling literature should consider including high-quality grey literature as part of their search, especially those concerned with gambling policy or interventions.

This study looked at only three countries. Since articles were assigned to the first author from one of the three target countries, this does not account for studies with primary authorship from another country (e.g. first author from the UK and second author from Canada), nor does it recognise collaborations between the target countries (e.g. first author from Australia and second author from New Zealand). The three countries investigated do not represent the full scope of gambling research and policy globally. We expect that if other major producers of gambling research were included (e.g. the UK or USA), new trends may be revealed as those countries have much larger proportions of research directly funded by the gambling industry. Future bibliometric investigations of the gambling studies literature could investigate work from more countries and examine the type(s) of gambling investigated, as well as the source of funding for each article. Although comprehensive typologies would have to be developed, this information could easily be coded from article abstracts and would provide important insights into the research interests of different jurisdictions, and how research trends are influenced by funders.

## Conclusions

In this study, we mapped 10 years of gambling research from Canada, Australia, and New Zealand to an established framework of harmful gambling, and examined the extent to which this research focused on gambling harm. Across the three countries, we observed an increasing trend in examining gambling harm over the 10-year period, and we found a clear dominance of research on the psychological factors of harmful gambling throughout the period. This matches previous findings and criticisms that gambling research is dominated by psychological and medical disciplines [14, 15, 55].

Within the countries examined, we found that research from New Zealand, with its public health model of gambling regulation, was the most focused on gambling environment and gambling resources and was most often concerned with harm. In Australia, with its privately operated gambling, the research was most often focused on issues of gambling exposure such as advertising and accessibility of gambling and was also often specifically concerned with harm. In Canada, a biopsychosocial lens on problem gambling may have created a focus on psychological and biological factors of harmful gambling and less explicit concern with gambling harm.

Although the links to policy seemed evident, it is worth remembering that although research funding opportunities are often linked to policy priorities, they will also reflect the mandates and funding priorities of support organisations such as VRGF in Australia and, in the past, OPGRC in Canada. Further, certain factors may continue to dominate because researchers who are able to obtain substantial funding are in a better position to attract graduate students and postdoctoral fellows who can provide the necessary support to continue their research programmes. In turn, these junior scholars develop expertise in the same area as their supervisor and can perpetuate this line of inquiry.

These findings provide important preliminary evidence that the modes of inquiry of gambling research are shaped by how gambling is regulated in the jurisdiction. In particular, we found that the jurisdiction with government-operated gambling, and no legislated requirement to focus on public health or harm minimisation, was highly focused on factors of harmful gambling that are the individual gamblers’ responsibility. Those who shape gambling research agendas should be aware of how the regulatory model of their jurisdiction may influence research questions and ensure that the harm from gambling is adequately investigated from diverse perspectives. Policy-makers and gambling researchers alike could benefit from an awareness of gambling research that is outside familiar disciplines and uses different methodological approaches in order to have a more complete view of how gambling is investigated and understood.

Future reviews of gambling harm research might investigate more forms of evidence, including grey literature, from more countries to find if these results are consistent cross-nationally. It would also be useful to continue monitoring trends over time that could be linked to policy mandates. Further investigations could also examine what is meant by “harm” in gambling research, in order to work towards a shared understanding of gambling harm within the field that aims to address it.

## Abbreviations

AGRC: Australian Gambling Research Centre
CFHG: Conceptual Framework of Harmful Gambling
CPGI: Canadian Problem Gambling Index
EGM: Electronic Gambling Machine
GREO: Gambling Research Exchange Ontario
MeSH: Medical Subject Headings
OPGRC: Ontario Problem Gambling Research Centre
PGSI: Problem Gambling Severity Index
TAB: Totalisator Agency Board
VLT: Video Lottery Terminal
WoS: Web of Science
